# Predictive value of PIMREG in the prognosis and response to immune checkpoint blockade of glioma patients

**DOI:** 10.3389/fimmu.2022.946692

**Published:** 2022-07-15

**Authors:** Hua Zhu, Xinyao Hu, Shi Feng, Lijuan Gu, Zhihong Jian, Ning Zou, Xiaoxing Xiong

**Affiliations:** ^1^ Department of Neurosurgery, Renmin Hospital of Wuhan University, Wuhan, China; ^2^ Department of Neurosurgery, The Affiliated Huzhou Hospital, Zhejiang University School of Medicine (Huzhou Central Hospital), Huzhou, China; ^3^ Cancer Center, Renmin Hospital of Wuhan University, Wuhan, China; ^4^ Central Laboratory, Renmin Hospital of Wuhan University, Wuhan, China; ^5^ Department of Radiation Oncology, Hubei Cancer Hospital, Tongji Medical College, Huazhong University of Science and Technology, Wuhan, China

**Keywords:** PIMREG, glioma, immune cell infiltrates, immune checkpoint, immune checkpoint blockade (ICB) therapy

## Abstract

Glioma is the most common primary brain tumor in the human brain. The present study was designed to explore the expression of PIMREG in glioma and its relevance to the clinicopathological features and prognosis of glioma patients. The correlations of PIMREG with the infiltrating levels of immune cells and its relevance to the response to immunotherapy were also investigated. PIMREG expression in glioma was analyzed based on the GEO, TCGA, and HPA databases. Kaplan–Meier survival analysis was used to examine the predictive value of PIMREG for the prognosis of patients with glioma. The correlation between the infiltrating levels of immune cells in glioma and PIMREG was analyzed using the CIBERSORT algorithm and TIMRE database. The correlation between PIMREG and immune checkpoints and its correlation with the patients’ responses to immunotherapy were analyzed using R software and the GEPIA dataset. Cell experiments were conducted to verify the action of PIMREG in glioma cell migration and invasion. We found that PIMREG expression was upregulated in gliomas and positively associated with WHO grade. High PIMREG expression was correlated with poor prognosis of LGG, prognosis of all WHO grade gliomas, and prognosis of recurrent gliomas. PIMREG was related to the infiltration of several immune cell types, such as M1 and M2 macrophages, monocytes and CD8+ T cells. Moreover, PIMREG was correlated with immune checkpoints in glioma and correlated with patients’ responses to immunotherapy. KEGG pathway enrichment and GO functional analysis illustrated that PIMREG was related to multiple tumor- and immune-related pathways. In conclusion, PIMREG overexpression in gliomas is associated with poor prognosis of patients with glioma and is related to immune cell infiltrates and the responses to immunotherapy.

## Introduction

Glioma is the most common primary brain tumor, and its 5-year survival rate is under 10% (1). Gliomas can be classified as grade I-IV based on the World Health Organization (WHO), of which WHO grade I-III is lower-grade glioma (LGG), and WHO IV is glioblastoma multiforme (GBM). The gold standard for the treatment of GBM is total resection plus radiation therapy, but the survival after diagnosis is poor. For LGG, surgical resection of the tumor combined with chemotherapy and radiotherapy improves the prognosis, but more than 50% of LGG patients eventually develop highly aggressive glioma (2). Therefore, identifying new therapeutic targets is crucial for finding new treatments for gliomas.

Phosphatidylinositol binding clathrin assembly protein interacting mitotic regulator (PIMREG, also referred to as CATS, RCS1, and FAM64A) has been verified to regulate the transition from mid to late cell division and is a biomarker of proliferation, suggesting an action in the development of cancer cells (3–5). Additionally, it was previously reported that PIMREG is expressed at high levels in lymphoma and leukemia cells (6). PIMREG has been reported to be a proliferation marker that promotes cholangiocarcinoma invasion by modulating the cell cycle (5). Additionally, high PIMREG expression can be considered a risk factor for the prognostic deterioration of pancreatic cancer (7). PIMREG has been reported to be associated with survival in prostate cancer (8). However, the oncogenic role of PIMREG in glioma has not been fully explored.

A crucial component of the tumor microenvironment is tumor-infiltrating immune cells (TIICs), which monitor tumor cells (9). Various immune components have been found in the glioma microenvironment, such as neutrophils, NK cells, macrophages, CD4+ helper T cells, and CD8+ cytotoxic T cells (10, 11). TIICs in the tumor microenvironment also influence tumor prognosis; for example, patients with thymoma with high infiltrating levels of B, CD4+ and dendritic cells (DCs) have a better prognosis and may be partially regulated by the ASF1B gene (12). Patients with LGG with high TUBA1C expression may have a sensitive response to immune checkpoint blockade (ICB) therapy (2). In the tumor microenvironment, lymphocytes represented by CD4+ Th cells, CD8+ cytotoxic T cells and CD4+/CD25+/FoxP3+ Tregs are important components of the immune response (13–15). The number of CD8+ cytotoxic T cells and CD4+ Th cells increases with tumor malignancy (11). Moreover, increased CD8+ cytotoxic T cell counts have been verified to be associated with improved patient prognosis (16). Upregulated programmed cell death ligand 1 (PD-L1) in glioma cells can block T cell activation and stimulate T cell apoptosis by binding to programmed death ligand 1 (PD-1), a suppressive immune checkpoint (17, 18). Furthermore, deficiency of CD80/86 costimulatory molecules leads to upregulation of CTLA-4, a strong inhibitor of CD4+ T cells and CD8+ cytotoxic T cells (11). In the early stages of glioma, tumor-associated macrophages suppress the proliferation of tumors through the “M1” proinflammatory phenotype, whereas in the late stages of glioma, tumor-associated macrophages are mainly characterized by “M2” macrophages, which usually cause immunosuppressive responses and immune escape of tumor cells (1). In recent years, neutrophils have been verified to enhance the progression and metastasis of tumors (19–21). Therefore, exploring targets associated with various immune cell infiltrations is of great importance for glioma treatment.

Among immunotherapies, ICB is the most widely employed immunotherapy for glioma in clinical practice. Through the binding of checkpoint molecules and particular antibodies, effector T cells can be reactivated and exert cytotoxic effects. T cell receptor-mediated signaling pathways can be negatively regulated by PD-1. By binding to PD-L1, PD-1 suppresses the activation and cytotoxic effects of T cells, blocking inflammatory factor production and leading to T cell inactivation. In gliomas, PD-L1 is mainly expressed on tumor cells and tumor-associated macrophages and negatively correlates with patient prognosis (22, 23). Anti-PD-L1 treatment leads to an elevated ratio of CD8+ cytotoxic T cells and Tregs. The efficacy of anti-PD-L1 therapy can be improved when used in combination with radiotherapy and other checkpoint inhibitors (24, 25). In addition, immune checkpoints, such as lymphocyte-activation gene 3 (LAG-3) and cytotoxic T-lymphocyte associated protein 4 (CTLA-4), are also important targets for ICB therapy (26, 27). However, the heterogeneity of tumors, alterations in checkpoints and widespread immunosuppression in the tumor microenvironment complicate glioma treatment. Therefore, promising preclinical studies are rarely successfully translated into clinical applications. Given this, it is critical to individualize therapy and monitor treatment response in real time. The prediction and monitoring of patient response to clinical immunotherapy has become an urgent requirement. Therefore, the search for biomarkers that predict immunotherapy for glioma is particularly important.

In the present study, we aimed to explore the prognostic role of PIMREG in glioma. The correlations of PIMREG with immune cell infiltration and response of glioma patients to ICB therapy were also studied.

## Methods

### Data acquisition

We obtained three glioma datasets from the GEO database (https://www.ncbi.nlm.nih.gov/geo/): GSE16011 (containing 8 normal samples and 276 glioma samples), GSE14805 (containing 4 normal samples and 32 glioma samples), and GSE19728 (containing 4 normal samples and 17 glioma samples). Gene sequence data of 662 gliomas (509 LGG and 153 GBM) and clinical information of patients were obtained from the TCGA database (https://cancergenome.nih.gov/).

### Analysis of PIMREG expression in gliomas

We analyzed the data downloaded from the GEO database through R software (version 4.0.3) and ggplot2 to explore the expression levels of PIMREG in glioma and normal brain tissues. Additional analysis was performed in GEPIA 2.0 (http://gepia2.cancer-pku.cn/#index) to evaluate PIMREG expression in normal brain tissue, LGG and GBM. We also analyzed 509 LGG samples and 153 GBM patient data in the TCGA database (https://portal.gdc.cancer.gov/) to compare the expression levels of PIMREG in different WHO classifications. Finally, the CGGA database (http://www.cgga.org.cn/) was also employed to explore PIMREG expression in glioma with different degrees of malignancy and in different WHO classifications, as well as the correlation with 1p/19q codeletion status and IDH mutation status.

### Analysis of PIMREG protein expression levels in gliomas

Immunohistochemical methods were used to assess PIMREG protein expression in glioma tissues and normal tissues to assess differences in protein levels. Three immunohistochemical images of normal cerebral cortex, three immunohistochemical images of low-grade glioma, and three immunohistochemical images of high-grade glioma were randomly obtained in the HPA database (http://www.proteinatlas.org/). The antibody used was an anti-PIMREG primary antibody (HPA 043783).

### Analysis of the correlation between PIMREG and glioma prognosis

To explore whether PIMREG could be regarded as an independent prognostic factor, univariate and multivariate Cox regression analyses were used. Clinical factors involved in Cox regression analyses included WHO grades, IDH status, sex, 1p/19q codeletion, and age. Using the R package ‘rms’, a nomogram and calibration were generated. To predict the 1-, 3-, and 5-year overall survival (OS), the ‘survival’ package was used. ROC analysis was performed using the ‘pROC’ R package to obtain the AUC curve.

The correlation between PIMREG and the OS and disease-free survival (DFS) of LGG and GBM was analyzed using the GEPIA 2.0 database. In addition, the CGGA database was employed to analyze the correlation between the survival of all WHO grade glioma patients and the survival of recurrent gliomas with PIMREG.

### Analysis of the correlation between PIMREG and the immune microenvironment and immune cell infiltration

We analyzed the levels of immune cell infiltrates in glioma by the TIMER database. In addition, the ESTIMATE algorithm and R package ‘limma’ were employed to evaluate the immune and stromal scores. The correlation between PIMREG and TIIC infiltration was analyzed as previously described (28).

### Analysis of the correlation between PIMREG and immune-related genes

The expression of eight immune checkpoint genes, SIGLEC15, TIGIT, CD274, HAVCR2, PDCD1, CTLA4, LAG3 and PDCD1LG2, was assessed in the high and low PIMREG expression groups using the R packages ‘ggplot2’ and ‘pheatmap’. In addition, the GEPIA 2.0 database was employed to analyze the relationship of PIMREG with these 8 immune checkpoint genes. We further analyzed the coexpression of PIMREG with other immune checkpoints and immune-related genes as we previously reported (29).

### Analysis of the correlation between PIMREG and response to ICB treatment

The Tumor Immune Dysfunction and Exclusion (TIDE) is an algorithm predicting the ICB response of individual samples. The TIDE algorithm was employed to evaluate the potential immunotherapeutic responses as we previously reported (29, 30). IMvigor210 cohorts that had received anti-PDL1 treatment were downloaded to verify the immunotherapy response prediction value of PIMREG as previously reported (31).

### Protein–protein interaction network construction

The STRING database (https://cn.string-db.org/) is an objective and extensive global network designed to collect, integrate and score published PPI information and complement these data with scientific calculations and predictions. The GeneMANIA (https://genemania.org/) database is a flexible and easy-to-use web-based tool for formulating gene function hypotheses, ranking genes for functional analysis, and generating lists of genes for analysis. It can discover and predict proteins with similar functions based on large amounts of genomic and proteomic data. The PPI network of PIMREG was investigated based on two online tools, STRING and GeneMANIA.

### KEGG pathway enrichment analysis and GO functional annotation analysis

The LinkedOmics database (http://www.linkedomics.org/login.php) is a public website containing multiple recombinant chemical data for 32 TCGA cancer types, including data from clinical proteomics tumor analysis. The potential biological functions of PIMREG in glioma were predicted using LinkedOmics, and KEGG pathway enrichment analysis and biological process GO analysis were completed by the gene set enrichment analysis (GSEA) method. In addition, the significantly related genes with PIMREG were analyzed by LinkedOmics.

### Validation of the role of PIMREG in glioma *in vitro*

The U251 and U87MG cell lines were used to perform transfection, qPCR, cell viability, wound healing, and transwell assays. The siRNA-PIMREG and siRNA-control were designed by GenePharma (Shanghai, China). The transfection was performed as previously described (9). qRT–PCR was performed 48 hours after transfection to evaluate the mRNA expression of PIMREG as previously reported (9). The primer sequences were PIMREG, F: GTGCTTTGGGTGCCGTGTC, R: ATCGCCGTAATGGGTGGG; GAPDH, F: GCACCGTCAAGGCTGAGAAC, R: TGGTGAAGACGCCAGTGGA. The viability of U87MG and U251 cells was evaluated using a CCK-8 kit. The role of PIMREG in the migration and invasion of glioma cells was explored by wound healing and transwell assays (9).

### Statistical analysis

Comparison of PIMREG expression differences in normal tissues and gliomas in the GEO-acquired dataset was performed using Wilcoxon tests. The Kruskal–Wallis test was employed to compare PIMREG expression differences in different WHO-graded gliomas in the TCGA-acquired data. Kaplan–Meier curves were applied to analyze the relationship between survival time and PIMREG expression levels. The correlation between PIMREG and immune checkpoints was analyzed by Spearman correlation analysis. Pearson correlation analysis was employed for KEGG pathway enrichment and GO function annotation analysis. The experimental data are presented as the means ± SD, and a two-group t test was run to compare the two groups. *P* < 0.05 was considered significantly different and is indicated by “*”.

## Results

### Differential expression of PIMREG in glioma and normal brain tissues

**Figure 1:**
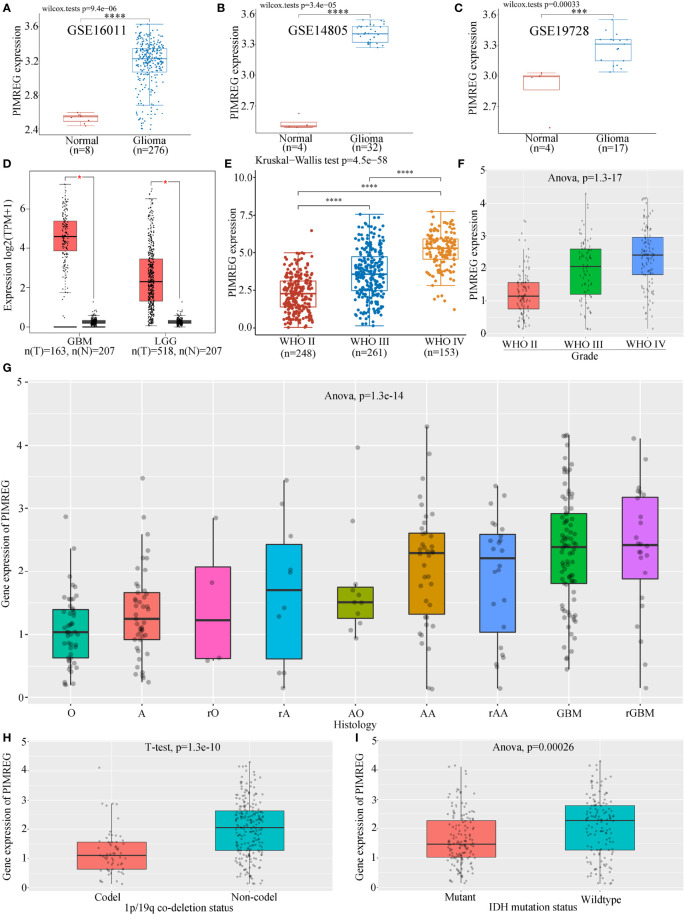
Differential expression of PIMREG in glioma and normal tissues. PIMREG expression was higher in glioma than in normal brain tissue in the GSE16011 **(A)**, GSE14805 **(B)**, and GSE19728 **(C)** datasets. **(D)** PIMREG expression was higher in both LGG and GBM than in normal brain tissue in the GEPIA 2.0 database. PIMREG expression analysis results from data downloaded from TGCA **(E)** and CGGA **(F)**. **(G)** Correlation between PIMREG expression in gliomas and the histology of gliomas. O: oligodendroglioma; A, astrocytoma; rO, recurrent oligodendroglioma; rA, recurrent astrocytoma; AO, anaplastic oligodendroglioma; AA, anaplastic astrocytoma; rAA, recurrent anaplastic astrocytoma; rGBM, recurrent glioblastoma. **(H)** PIMREG expression in gliomas with 1p/19q codeletion and non1p/19q codeletion. **(I)** Expression of PIMREG in IDH-mutant gliomas and wild-type gliomas. *P < 0.05; ***P < 0.001; ****P < 0.0001.

**Figure 2:**
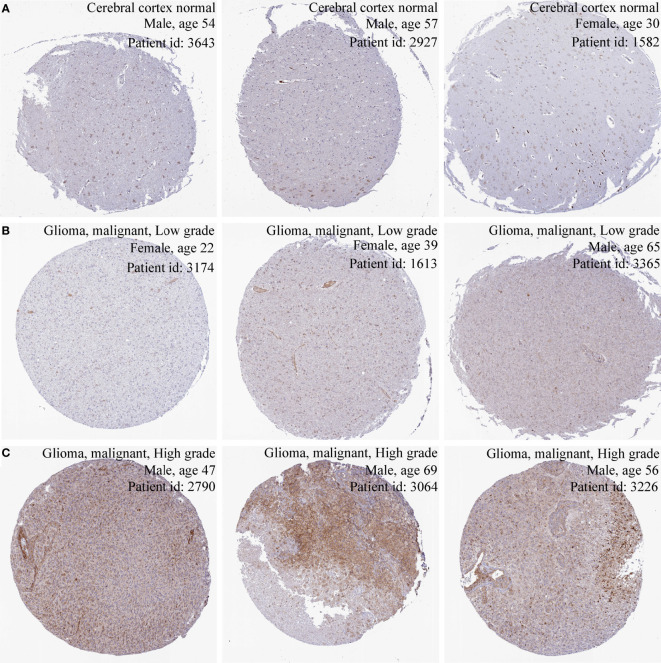
Immunohistochemical images of PIMREG in glioma. Immunohistochemical images of PIMREG in normal brain tissue **(A)**, LGG **(B)**, and GBM **(C)**.

The results of three datasets obtained from the GEO database, GSE16011 (*P* = 9.4e-06) (Figure 1A), GSE14805 (*P* = 3.4e-05) (Figure 1B), and GSE19728 (*P* = 0.00033) (Figure 1C), demonstrated that PIMREG expression was upregulated in gliomas compared to normal brain tissues. The results from GEPIA 2.0 analysis revealed higher expression of PIMREG in LGG (518 cases) and GBM (163 cases) than in normal tissue (207 cases) (*P* < 0.05) (Figure 1D). Analysis of the data downloaded from TCGA further illustrated that PIMREG expression was highest in WHO grade IV and lowest in WHO grade II (Figure 1E). Similar results were observed in the CGGA database (Figure 1F). In addition, PIMREG expression correlated with glioma histology, for example, low expression in oligodendroglioma and astrocytoma and high expression in glioblastoma and secondary glioblastoma (Figure 1G). PIMREG expression was low in glioma with combined 1p/19q deletion (*P* = 1.3e-10) (Figure 1H) and in IDH-mutated glioma (*P* = 0.00026) (Figure 1I). Immunohistochemical images of PIMREG obtained from the HPA database of normal brain tissue (Figure 2A), LGG (Figure 2B), and GBM (Figure 2C) are presented in Figure 2.

### Correlation of PIMREG expression with gliomas on patient prognosis

**Figure 3:**
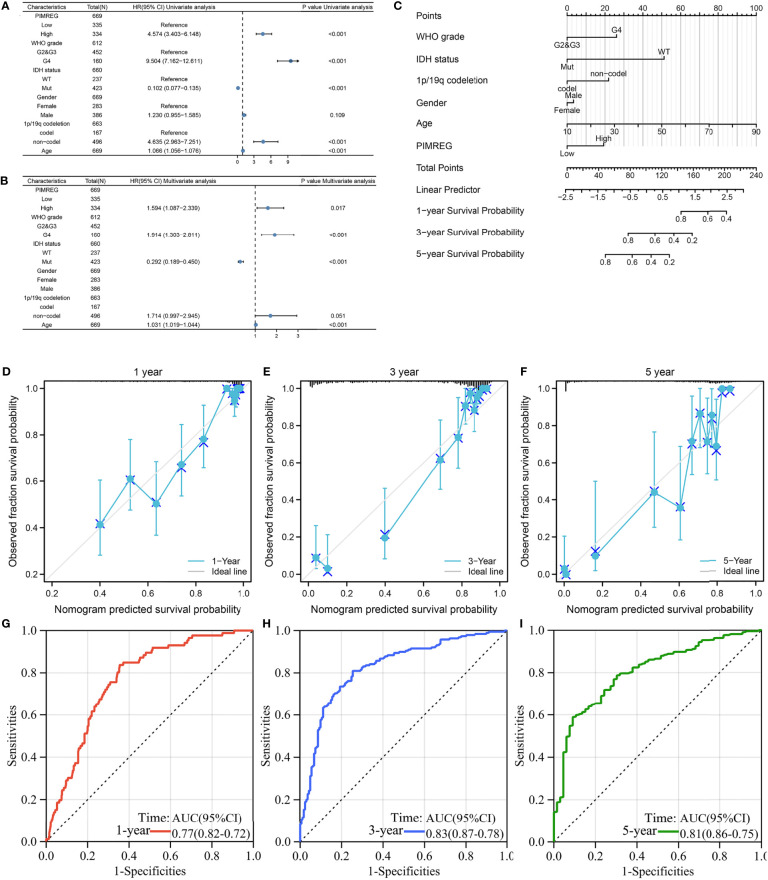
The prognostic value of PIMREG in glioma. **(A, B)** Univariate and multivariate Cox regression visualized in the forest plot. **(C)** Nomogram and calibration plots **(D–F)** predicting the 1-year, 3-year and 5-year OS of glioma patients. **(G–I)** Predictive ability for 1-, 3-, and 5-year prognosis with PIMREG expression by time-dependent ROC curve analysis.

Univariate Cox regression illustrated that PIMREG expression, WHO stage, 1p/19q codeletion, age and IDH status were associated with the prognosis of glioma (Figure 3A). Multivariate Cox regression analysis demonstrated that PIMREG expression (*P* = 0.017), WHO grade (*P* < 0.001), IDH status (*P* < 0.001), and age (*P* < 0.001) were independent prognostic factors for the prognosis of gliomas (Figure 3B). A nomogram was constructed to provide a quantitative guideline to predict 1-, 3-, and 5-year OS in patients with glioma (Figure 3C). Moreover, the calibration curves demonstrated that the nomogram was able to accurately estimate 1-, 3-, and 5-year OS (**Figures 3D–F**). Time-dependent ROC analysis showed that the AUC values for 1, 3, and 5 years were 0.77, 0.83, and 0.81 in glioma, respectively, indicating a high predictive power (**Figures 3G–I**). PIMREG can therefore be used as a possible diagnostic marker for glioma.

**Figure 4:**
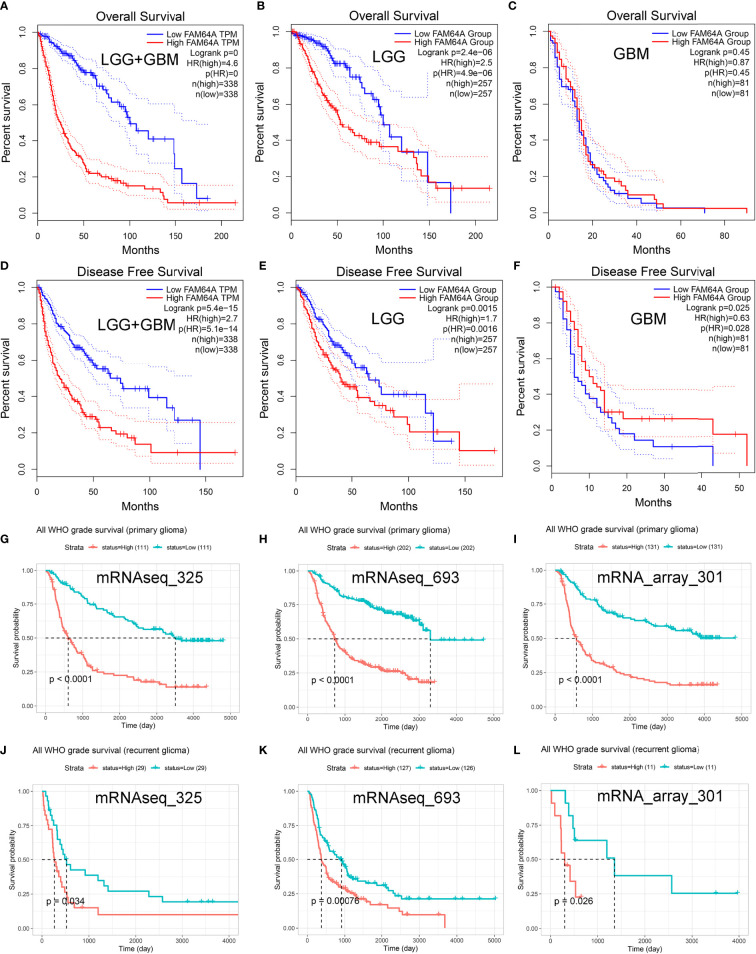
Predictive value of PIMREG (FAM64A) in glioma prognosis. Correlation of PIMREG expression with OS of glioma **(A)**, LGG **(B)**, and GBM **(C)**. Correlation of PIMREG with the DFS of glioma **(D)**, LGG **(E)** and GBM **(F)**. Correlation between PIMREG and survival probability of all WHO-grade primary **(G–I)** and recurrent **(J–L)** glioma in CGGA datasets.

Glioma patients who had high expression of PIMREG showed poorer OS (*P* < 0.0001) (Figure 4A) and DFS (*P* = 5.4e-14) (Figure 4D) than those with low expression of PIMREG. Patients with LGG with high PIMREG expression had poorer OS than those with low PIMREG expression (*P* = 2.4e-06) (Figure 4B). LGG patients with high PIMREG expression had worse DFS than those with low PIMREG expression (*P* = 0.0015) (Figure 4E). There was no significant difference in prognosis between the high and low PIMREG expression groups in GBM patients (*P* = 0.45) (Figure 4C), but patients with high PIMREG expression in GBM had better DFS than those with low expression (*P* = 0.025) (Figure 4F). In addition, we also obtained the effect of PIMREG on survival in all WHO-graded gliomas from the CGGA database, and we found that patients with gliomas with high PIMREG expression had a worse prognosis than those with low PIMREG expression (*P* < 0.0001) (**Figures 4G–I**); similar results were found in all WHO-graded secondary gliomas (**Figures 4J–L**).

### PIMREG promotes the migration and invasion of glioma cells

**Figure 5:**
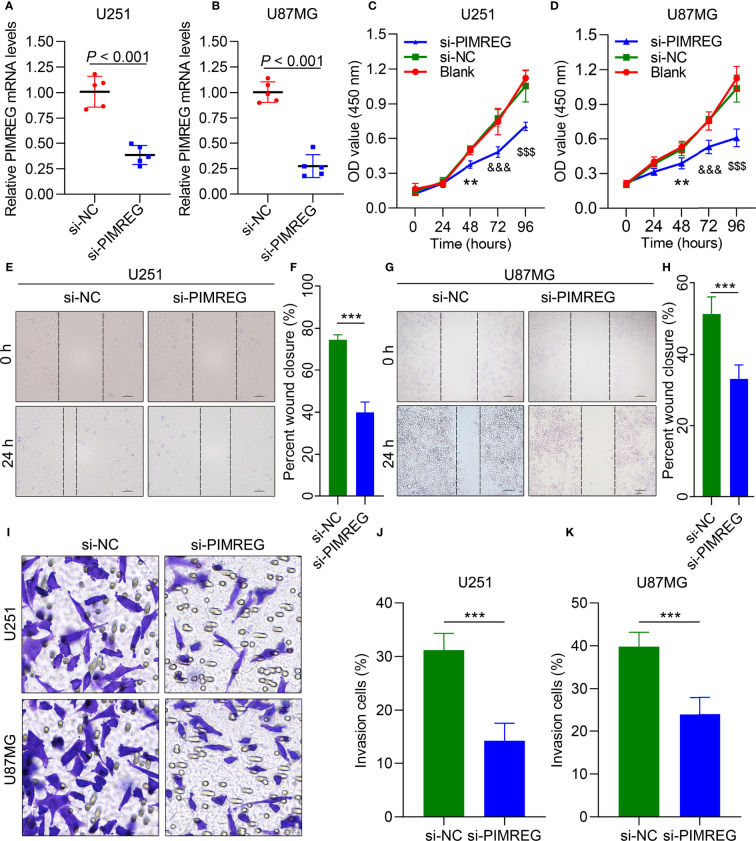
PIMREG enhances the migration and invasion of glioma cells *in vitro*. qRT–PCR results showing PIMREG expression in U251 **(A)** and U87MG **(B)** glioma cells after transfection. CCK-8 assays showing the viability of U251 **(C)** and U87 **(D)** cells. Wound healing was performed to determine the migration ability of U251 cells **(E, F)** and U87MG cells **(G, H)**. Transwell assays were performed to determine their invasion ability **(I–K)**. **P < 0.01; ***P < 0.001; $$$P < 0.001; &&&; P< 0.0001.

We further performed experiments *in vitro* to verify the role of PIMREG in glioma cells. mRNA expression was knocked down by siRNA-PIMREG in U251 (*P* < 0.001) (Figure 5A) and U87MG (*P* < 0.001) (Figure 5B) cells. The viability of glioma cells was inhibited by siRNA-PIMREG (**Figures 5C, D**). Wound healing assays demonstrated that siRNA-PIMREG suppressed the wound closure percent compared with siRNA-control (**Figures 5E–H**). The transwell assay indicated that PIMREG knockdown inhibited the invasion of U251 and U87MG glioma cells (**Figures 5I–K**). These results demonstrated that PIMREG knockdown significantly suppressed the migration and invasion of U251 and U887MG cells.

### Correlation of PIMREG expression with glioma immune cell infiltrates

**Figure 6:**
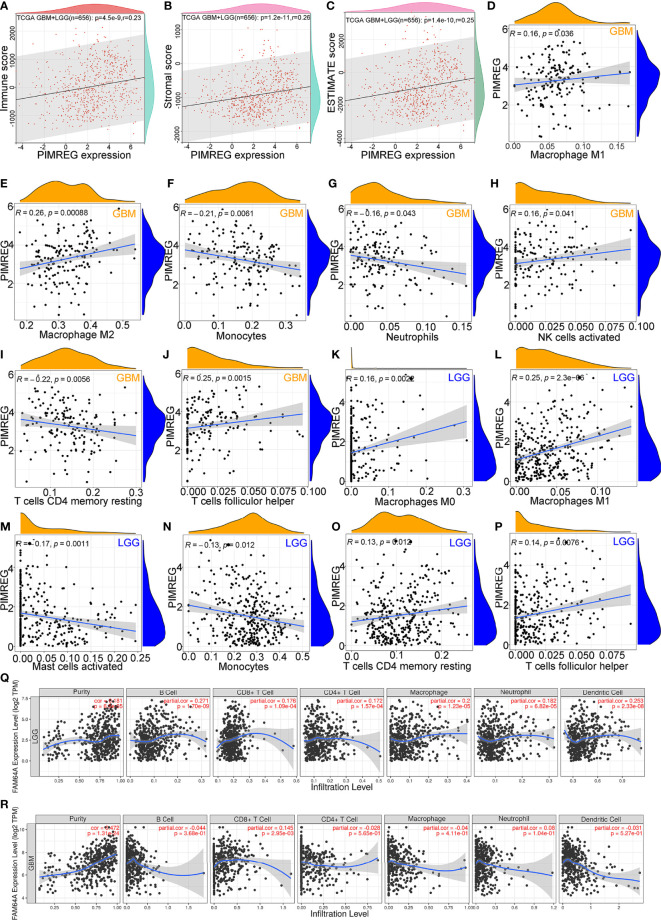
Correlation between PIMREG expression and tumor immunity of gliomas. Correlation of PIMREG with immune score **(A)**, stromal score **(B)** and ESTIMATE score **(C)** in LGG+GBM. Correlation of PIMREG with the infiltrating levels of M1 macrophages **(D)**, M2 macrophages **(E)**, monocytes **(F)**, neutrophils **(G)**, activated NK cells **(H)**, resting memory CD4 T cells **(I)**, and follicular helper T cells **(J)** in GBM. Correlation of PIMREG with the infiltrating level of M0 macrophages **(K)**, M1 macrophages **(L)**, activated mast cells **(M)**, monocytes **(N)**, resting memory CD4 T cells **(O)**, and follicular helper T cells **(P)** in LGG. Correlation between PIMREG and immune cells in LGG **(Q)** and GBM **(R)** in the TIMER database.

PIMREG expression was positively correlated with (LGG+GBM) immune scores (*P* = 4.5e-9, Cor = 0.23) (Figure 6A), stromal scores (*P* = 1.2e-11, Cor = 0.26) (Figure 6B), and ESTIMATE scores (*P* = 1.4e-10, Cor = 0.25) (Figure 6C). We employed CIBERSORT to analyze the correlation between PIMREG expression and the degree of immune cell infiltrates in GBM and LGG. The results showed that PIMREG expression in GBM was positively related to, M2 macrophages (*P* = 0.00088, Cor = 0.26) (Figure 6E), and follicular helper T cells (*P* = 0.0015, Cor = 0.25) (Figure 6J). The expression of PIMREG in GBM was negatively associated with the infiltration of monocytes (*P* = 0.0061, Cor = -0.21) (Figure 6F), and resting CD4 memory cells (*P* = 0.0056, Cor = -0.22) (Figure 6I). The correlations of PIMREG with M1 macrophages (*P* = 0.036, Cor = 0.16) (Figure 6D), neutrophils (*P* = 0.043, Cor = -0.16) (Figure 6G), activated NK cells (*P* = 0.041, Cor = 0.16) (Figure 6H) were extremely weak in GBM. In LGG, PIMREG expression was positively related to M1 macrophages (*P* = 2.3e-06, Cor = 0.25) (Figure 6L).

However, the correlations of PIMREG with M0 macrophages (*P* = 0.0022, Cor = 0.16) (Figure 6K), resting CD4 memory cells (*P* = 0.0012, Cor = 0.13) (Figure 6O), follicular helper T cells (*P* = 0.0076, Cor = 0.14) (Figure 6P), activated mast cells (*P* = 0.0011, Cor = -0.17) (Figure 6M) and monocytes (*P* = 0.0012, Cor = -0.13) (Figure 6N) were extremely weak. In addition, we analyzed the infiltration of other immune cell types by the TIMER database. The findings showed that PIMREG expression was positively correlated with tumor purity in LGG (*P* = 6.5e-05, Cor = 0.181) (Figure 6Q) and GBM (*P* = 1.31e-24, Cor = 0.472) (Figure 6R). In LGG, the expression of PIMREG was positively related to the infiltrating levels of macrophages (*P* = 1.23e-05, Cor = 0.2), B cells (*P* = 1.70e-09, Cor = 0.271), and DCs (*P* = 2.33e-08, Cor = 0.253). In GBM, PIMREG expression was weakly positively related to CD8+ T cell infiltrates (*P* = 2.95e-03, Cor = 0.145) (Figure 6R).

### Predictive value of PIMREG in the response of glioma patients to ICB

**Figure 7:**
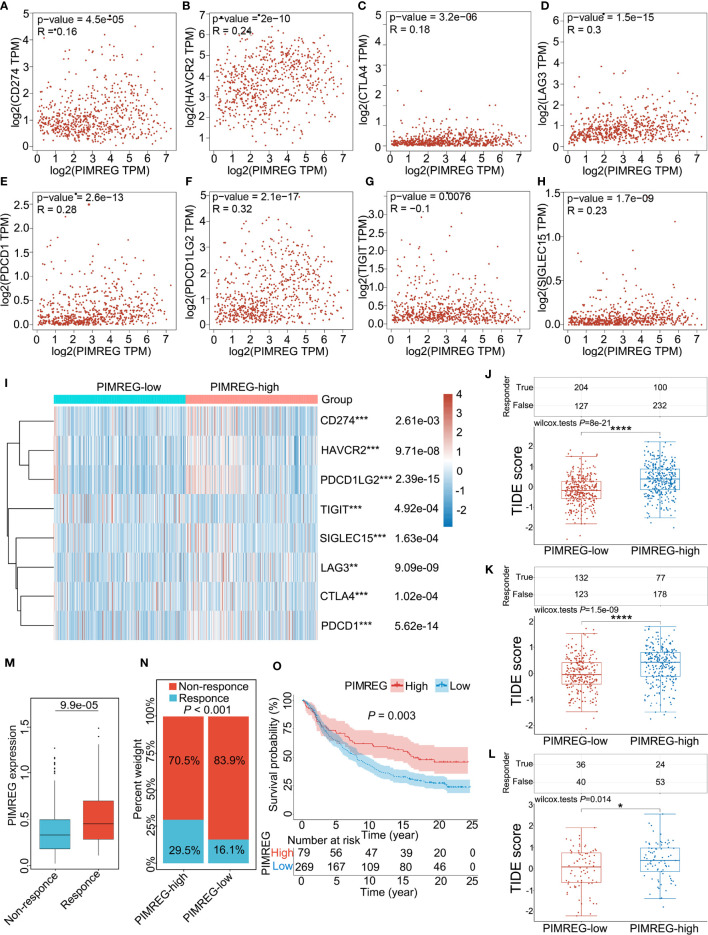
Correlation between PMREG and immune checkpoints and response to immunotherapy. Correlation of PIMREG with the immune checkpoints CD274 **(A)**, HAVCR2 **(B)**, CTLA4 **(C)**, LAG3 **(D)**, PDCD1 **(E)**, PDCD1LG2 **(F)**, TIGIT **(G)**, and SIGLEC15 **(H)** in gliomas (LGG+GBM). **(I)** Heatmap of immune checkpoint expression in gliomas with high and low PIMREG expression (LGG+GBM). **(J)** Different TIDE scores in gliomas (LGG + GBM) with high and low expression of PIMREG. **(K)** Different TIDE scores in LGG with high and low expression of PIMREG. **(L)** Different TIDE scores in GBM with high and low expression of PIMREG. **(M)** PIMREG expression in the response and nonresponse groups to anti-PD-1 therapy in the IMvigor210 cohort (anti-PD-L1, urological). **(N)** The ratio of patients who responded to anti-PD-1 therapy in the low- and high-PIMREG groups of the IMvigor210 cohort. **(O)** Kaplan–Meier curves for the low- and high-PIMREG patient groups in the IMvigor210 cohort. *P < 0.05; **P < 0.01; ***P < 0.001; ****P < 0.0001.

Glioma (LGG+GBM) samples downloaded from the TCGA database were divided into PIMEG-low and PIMREG-high groups according to PIMREG expression (high and low expression levels are classified by the median), and we revealed that immune checkpoint genes (including CD274, HAVCR2, PDCD1LG2, SIGLEC15, LAG3 CTLA4, and PDCD1) were overexpressed compared with the PIMREG-low expression group (*P* < 0.001) (Figure 7I), while TIGIT expression was lower in PIMREG high-expressing glioma patients than in PIMREG low-expressing glioma patients (*P* = 4.92e-04) (Figure 7I). In addition, we analyzed the relationship of PIMREG with these immune checkpoint genes in gliomas through the GEPIA 2.0 database. PIMREG expression in glioma was positively correlated with HAVCR2 (*P* = 2e-10, Cor = 0.24) (Figure 7B), LAG3 (*P* = 1.5e-15. Cor = 0.30) (Figure 7D), PDCD1 (*P* = 2.6e-13, Cor = 0.28) (Figure 7E), PDCD1LG2 (*P* = 2.1e-17, Cor = 0.32) (Figure 7F), and SIGLEC15 (*P* = 1.7e-09, Cor = 0.23) (Figure 7H). However, the correlations of PIMREG with CD274 (*P* = 4.5e-05, Cor = 0.16) (Figure 7A), CTLA4 (*P* = 3.2e-06, Cor = 0.18) (Figure 7C), and TIGIT (*P* = 0.0076, Cor = -0.10) (Figure 7G) were extremely weak.

Additionally, we evaluated the correlation of PIMREG expression with the intensity of the response to immunotherapy in glioma patients. The results indicated that, in glioma (LGG+GBM), patients with high PIMREG expression had higher TIDE scores than those with low PIMREG expression (*P* = 8e-21) (Figure 7J). In LGG, TIDE scores were higher in glioma patients with high PIMREG expression than in patients with low PIMREG expression (*P* = 1.5e-09) (Figure 7K). We also analyzed similar results in GBM (*P* = 0.014) (Figure 7L). These findings suggest a correlation of PIMREG with immune checkpoints in gliomas and that glioma patients with high PIMREG expression may be more sensitive to immunotherapy.

Additionally, in the urological tumors of the IMvigor210 cohort, PIMREG expression was significantly higher in the ICB-responsive group than in the nonresponsive group (*P* = 9.9e-05) (Figure 7M). The response ratio to anti-PD-L1 therapy was 29.5% in high-PIMREG expression patients and >16.1% in low-PIMREG expression patients (Figure 7N). Moreover, the PIMREG-high group had a better survival probability than the PIMREG-low group (*P* = 0.003) (Figure 7O). These results confirmed that PIMREG is capable of predicting immunotherapy response.

### Coexpression of PIMREG with immune-related genes in glioma

**Figure 8:**
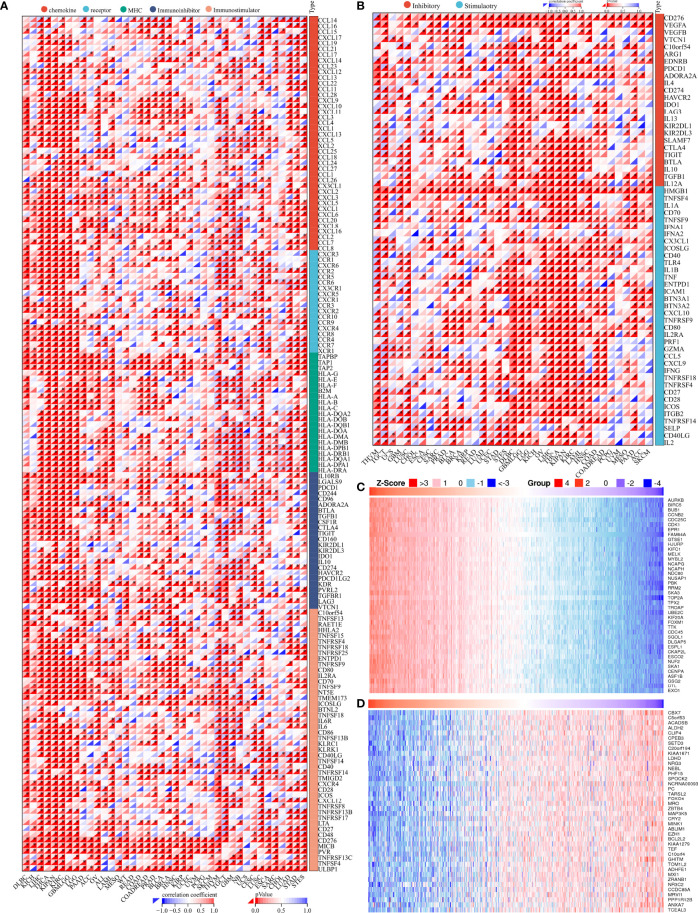
Genes coexpressed with PIMREG. **(A)** Coexpression of PIMREG with immune-related genes. **(B)** Coexpression of PIMREG with inhibitory and stimulatory immune checkpoints. Significantly positively **(C)** and negatively **(D)** coexpressed genes with PIMREG. *P < 0.05.

To further investigate the role of PIMREG in the antitumor immunity of LGG and GBM, PIMREG was coexpressed with most chemokine (receptor), MHC, immunoinhibitory, and immunostimulatory genes in pancancer (28). Similar results were found in LGG and GBM (Figure 8A). Moreover, PIMREG was coexpressed with various inhibitory and stimulatory immune checkpoints (Figure 8B). Additionally, the top 40 positive and negative PIMREG coexpressed genes are shown in **Figures 8C, D**.

### PPI network, GO functional annotation and KEGG pathway enrichment analysis of PIMREG

**Figure 9:**
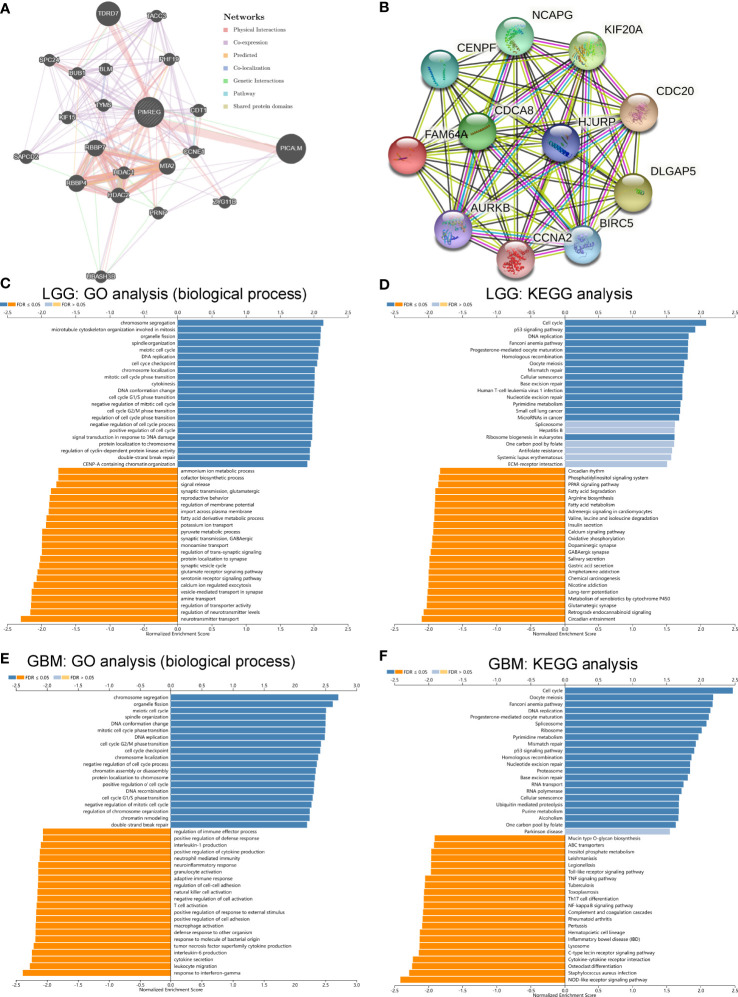
Protein–protein interaction network, GO and KEGG pathway enrichment analysis of PIMREG in glioma. PPI network of PIMREG in the GeneMINIA **(A)** and SRTING **(B)** tools. GO analysis (biological process) of PIMREG **(C)** and KEGG pathway enrichment analysis **(D)** in LGG in LinkedOmics. GO analysis (biological process) of PIMREG **(E)** and KEGG pathway enrichment analysis **(F)** in GBM in LinkedOmics.

As shown in Figure 9, we predicted the PPI network interacting with PIMREG by the GeneMINIA and STRING tools. The proteins that interacted with PIMREG by the GeneMINIA tool included TDRD7, PICALM, HDAC1, HDAAC2, RBBP4, MTA2, and CCNE1 (Figure 9A). The proteins predicted to interact with PIMREG by the STRING tool included CDCA8, CENPF, AURK8, CCNA2, BIRC5, KIF20A, and DLGAP5 (Figure 9B).

Analysis of the potential function of PIMREG in glioma was performed by the LinkedOmics tool. We analyzed the function of PIMREG in LGG, and GO biological process analysis showed that PMREG was associated with chromosome segregation, microtubule cytoskeleton organization involved in mitosis, organelle fission, cell cycle G1/S phase transition, and cell cycle checkpoint (Figure 9C). KEGG pathway enrichment analysis also showed that PIMREG in LGG was positively correlated with the P53 pathway, the cell cycle, DNA replication, and oocyte meiosis (Figure 9D). In GBM, GO biological process analysis showed that PIMREG was associated with chromosome segregation, organelle fission, meiotic cell cycle, DNA conformational changes, mitotic cell cycle phase changes, DNA replication, cell cycle G2/M phase changes, and other cell cycle change functions. The production of tumor necrosis factor superfamily cytokines, T cell activation, positive regulation of responses to external stimuli, positive regulation of cell adhesion, macrophage activation, adaptive immune response, cell adhesion regulation, NK cell activation, negative regulation of cell activation, T cell activation, neuroinflammatory responses, neutrophil-mediated immunity, production of interleukin 1, regulation of immune effector processes, and other processes were correlated (Figure 9E). In GBM, KEGG pathway enrichment analysis showed that PIMREG was associated with the cell cycle, P53 cell pathway, and oocyte meiosis and negatively associated with the NF-κB pathway, cytokine–cytokine receptor interaction, Toll-like receptor signaling pathway, Th17-cell differentiation, TNF pathway and other pathways (Figure 9F).

## Discussion

In this work, we found that PIMREG expression was upregulated in gliomas compared with normal brain tissues and increased with WHO grade. The results from the HPA database analysis illustrated that the PIMREG protein level was higher in GBM than LGG, while it was lowest in normal brain tissues. To some extent, the malignancy of glioma may be predicted by measuring the mRNA or protein content of PIMREG. Furthermore, in LGG patients with high PIMREG expression, the prognosis was worse than in patients with low PIMREG expression, suggesting that, in LGG, PIMREG predicts the OS and DFS of patients. In all WHO grade gliomas, the overall survival time was shorter in patients with high PIMREG expression than in those with low PIMREG expression. The same results were also found in secondary gliomas. This suggests that PIMREG may be a predictor of prognosis in all WHO-graded gliomas. However, no significant differences in OS were observed between GBM patients in the PIMREG-high and PIMREG-low groups. High malignancy of GBM combined with high PIMREG expression may be the two factors contributing to this result. In conclusion, there is a relationship between PIMREG expression and patient prognosis, which may be a predictor of prognosis in glioma patients.

TIICs, important components of the tumor microenvironment, have a dual function of immunostimulation or immunosuppression, promoting or inhibiting tumor development (32). The degree of infiltration of TIICs into the tumor microenvironment also influences tumor prognosis. In our study, we revealed a relationship between PIMREG expression in gliomas and the infiltration of some TIICs. The immune, stromal and ESTIMATE scores in gliomas were positively correlated with PIMREG expression. The findings showed that PIMREG expression in GBM positively correlated with the infiltrates of M1 and M2 macrophages, activated NK cells, and follicular helper T cells, and PIMREG expression in GBM negatively correlated with the infiltration of monocytes, neutrophils, and resting memory CD4 cells. In LGG, PIMREG expression was positively related to M0 macrophage, resting CD4 memory cell, and follicular helper T cell infiltration at a super high level of difficulty and negatively related to the infiltration of activated mast cells and monocytes. In conclusion, PIMREG correlates with the degree of partial immune cell infiltrates in glioma and may affect the prognosis of glioma patients by influencing the degree of immune cell infiltration.

Recently, ICB has been applied to glioma treatment, changing the paradigm of glioma treatment (33). Tumor cells tend to evade CTL destruction by upregulating immune checkpoint ligands (e.g., PD-L1) that can bind to complementary receptors (PD-1) on CTLs, leading to the suppression of lymphocyte activation. Except for PD-1, CTLA4, and LAG3, the expression of other immunosuppression-related genes, such as LGALS1 and IGFBP2, is higher in glioma patients, and blocking the expression of immunosuppression-related genes can reshape the immunosuppressive microenvironment (34, 35). However, the immunosuppressive microenvironment of tumors is one of the main reasons for chemoresistance and immunotherapy failure in patients with diffuse glioma. The heterogeneity of each patient and their different responses to immunotherapy make immunotherapy for glioma very difficult. Herein, we found that the expression of several immune checkpoints was higher in glioma patients with high PIMREG expression than in those with low PIMREG expression. PIMREG expression in glioma was positively correlated with these immune checkpoints. In addition, we assessed the relationship between PIMERG expression and the intensity of response to immunotherapy in glioma patients. The results indicated that, in gliomas, patients with high PIMREG expression had higher TIDE scores than those with low PIMREG expression. These results suggest that PIMREG correlates with most immune checkpoints in gliomas and that patients with gliomas with high PIMREG expression may be more sensitive to immunotherapy. Therefore, PIMREG may be a predictive marker for the intensity of the response to ICB in glioma patients.

We analyzed the function of PIMREG in LGG, and GO biological process analysis showed that PMREG was positively associated with chromosome segregation, organelle fission, cell cycle G1/S phase transition, and cell cycle checkpoint. KEGG pathway enrichment analysis also showed that PIMREG in LGG was positively associated with the P53 pathway, cell cycle, DNA replication, and oocyte meiosis. In GBM, GO biological process analysis showed that PIMREG was positively associated with chromosome segregation, organelle fission, meiotic cell cycle, DNA conformational changes, mitotic cell cycle phase changes, DNA replication, and cell cycle G2/M phase changes. PIMREG was associated with several immune-related biological processes in GBM, including leukocyte migration, cytokine secretion, interleukin 6 production, T cell activation, macrophage activation, adaptive immune response, cell adhesion regulation, NK cell activation, positive regulation of cell adhesion, activation of T cells, neuroinflammatory responses, negative regulation of cell activation, neutrophil-mediated immunity, production of interleukin 1, and immune effector process regulation. In GBM, KEGG pathway enrichment analysis showed that PIMREG was positively associated with the cell cycle, P53 cell pathway, and oocyte meiosis and negatively associated with the NOD-like receptor pathway, C-type lectin receptor pathway, NF-κB pathway, cytokine–cytokine receptor interaction, Toll-like receptor signaling pathway, Th17-cell differentiation, TNF pathway and other pathways. These results suggest that PIMREG promotes glioma development through numerous immune-related pathways or biological processes in addition to affecting the cell cycle of glioma cells.

## Conclusion

In conclusion, PIMREG was highly expressed in gliomas and correlated with WHO classification. High PIMREG expression correlated with poor prognosis of low-grade gliomas, poor prognosis of all WHO-graded gliomas, and poor prognosis of recurrent tumors. PIMREG expression in glioma was correlated with several immune cells. PIMREG correlated with CTLA-4, PDCD1, LAG3 and other immune checkpoints in glioma and correlated with the patient’s response to immunotherapy. PIMREG correlated with the cell cycle and immune-related pathways. PIMREG may be used as a prognostic marker in glioma and possibly as a biomarker of response to immunotherapy.

## Data availability statement

This data can be found here: GEO database (https://www.ncbi.nlm.nih.gov/geo/); TCGA database (https://cancergenome.nih.gov/); CGGA database (http://www.cgga.org.cn/); HPA database (http://www.proteinatlas.org/). The accession number(s) can be found in the article/supplementary material.

## Author contributions

HZ and SF analyzed the data. XX and NZ designed this study. XH and HZ wrote the article. HZ performed cell experiments. ZJ and LG revised the manuscript. All authors have read and agreed to the published version of the manuscript. All authors contributed to the article and approved the submitted version.

## Funding

This work was supported by the National Natural Science Foundation of China (No. 81870939 to Xiaoxing Xiong), the Fundamental Research Funds for the Central Universities (No.2042022kf1216 to Xiaoxing Xiong), the Natural Science Foundation of Hubei Province (No.2016CFB405 to Ning Zou) and Wuhan Young and Middle-aged Medical Backbone Talents Training Project in 2017 to Ning Zou.

## Conflict of interest

The authors declare that the research was conducted in the absence of any commercial or financial relationships that could be construed as a potential conflict of interest.

## Publisher’s note

All claims expressed in this article are solely those of the authors and do not necessarily represent those of their affiliated organizations, or those of the publisher, the editors and the reviewers. Any product that may be evaluated in this article, or claim that may be made by its manufacturer, is not guaranteed or endorsed by the publisher.
